# Incomplete oedipism and chronic suicidality in psychotic depression with paranoid delusions related to eyes

**DOI:** 10.1186/1744-859X-5-18

**Published:** 2006-11-21

**Authors:** Maurizio Pompili, David Lester, Roberto Tatarelli, Paolo Girardi

**Affiliations:** 1 McLean Hospital – Harvard Medical School, Boston, MA, USA; 2 Department of Psychiatry – Sant'Andrea Hospital, University of Rome "La Sapienza", Rome, Italy; 3 Center for the Study of Suicide, Blackwood, New Jersey, USA

## Abstract

Self-enucleation or oedipism is a term used to describe self-inflicted enucleation. It is a rare form of self-mutilation, found mainly in acutely psychotic patients. We propose the term incomplete oedipism to describe patients who deliberately and severely mutilate their eyes without proper enucleation.

We report the case of a 32-year-old male patient with a five-year history of psychotic depression accompanied by paranoid delusions centered around his belief that his neighbors criticized him and stared at him. A central feature of his clinical picture was an eye injury that the patient had caused by pouring molten lead into his right eye during a period of deep hopelessness and suicidality when the patient could not resolve his anhedonia and social isolation. Pharmacotherapy and psychotherapy dramatically improved his disorder.

## Background

Severe intentional eye self-injury is an uncommon, but not rare, condition. Such injuries have been documented primarily in Christian cultures [1]. Favazza [1] estimated that there were about 500 cases per year and, according to Favazza and Rosenthal's [2] criteria for self-mutilation, eye self-injury is considered to be a major self-mutilation. Abrasion or introduction of chemical substances into the conjunctival sac has been found in factitious disorder [3], malingering [4], and character pathology [5]. Rogers [6] has suggested that the severity of the eye injury produced is proportional to the severity of the psychopathology. Tapper et al. [7] reviewed the international literature from 1848 to 1957 and found 34 cases of severe, self-inflicted eye injury of which 27 had received a psychiatric diagnosis, including 9 patients with schizophrenia, 13 with affective disorder, and 4 with organic conditions.

Self-enucleation or oedipism (the act of destroying one or both eyes) has been described in psychotic patients [8], most frequently in schizophrenics [9]. Feldman and Feldman [10] reported that, after performing self-enucleation, patients were often found with a copy of Matthew's Gospel open at 5:29 where it is states "...if the right eye offend thee, pluck it out and cast it from thee; for it is profitable for thee that one of thy members should perish and not that thy whole body should cast into hell". Apparently, the enucleation enacts a literal interpretation of the text. Matthew's Gospel (5:28) also states that "everyone who has looked at a woman lustfully has already committed adultery with her in his heart," thereby making the act of looking a sin.

Cases of self-enucleation have also been described in patients with drug-induced psychosis [11], bipolar disorder [7], obsessive compulsive disorder [12], post traumatic stress disorder [13] and depression [14]. According to Moskovitz and Byrd [15] the following similarities are found in self-enucleation patients: the act is viewed as a means of saving themselves or the world; the patients do not regret the action; they often quoted biblical passages; and they were psychotic at the time of the act. MacLean and Robertson [16] reviewed the literature and noted that castration fears, failure to resolve oedipal conflicts, repressed homosexual impulses, severe guilt, and severe self-punishment were common psychodynamic features of these cases.

We report a case here of a young man who attempted to destroy his right eye. He had been suicidal for a number of years, was diagnosed as having a psychotic depression, had social phobia, somatic anxiety and compulsive obsessive traits, and showed perversion and delusions.

## Case history

Mr. C, aged 32, poured molten lead into his right eye during a period of great emotional distress and during a time when pharmacological treatment for his depression was not producing any beneficial effect. We call such an action incomplete oedipism since the patient did not enucleate the eye, but merely damaged it. After the injury, he was hospitalized for a long period and, after much medical treatment, had an almost normal eye. Destroying the eye was, according to his words, a way of blackmailing his parents. One evening, he had quarreled with his parents who had denied him permission to buy a motorbike. As a result, he decided to punish them by damaging his own eye. This action took place after a long history of psychiatric treatment, including prescription of a wide variety of psychotropic drugs, ranging from neuroleptics to antidepressants, as well as atypical antipsychotics.

He had experienced at least three previous depressive episodes but no hypomania. His first depressive episode was at the age of twenty. At time of our evaluation a DSM-IV diagnosis of major depression was made comorbid with DSM-IV-TR delusion disorder (persecutory type). One of his main symptoms was a paranoid delusion that other people, and in particular people living in his neighborhood, stared at him all the time and laughed at him. This belief made the patient angry and depressed since, as a result, he felt unable to leave his home and, in addition, he experienced great anxiety.

He grew up in a very disturbed family. His sister had a serious obsessive-compulsive disorder. His elderly parents lacked empathy and showed hysterical and obsessive behaviors. His relationship with his mother was very disappointing for him as she was emotionally distant. She would blackmail his father by pretending to faint and by lying on the floor as if dead. The father rejected his son, fearing that he could get infected by the patient. The father said that he had not wanted him, and he ignored the son.

The patient had experienced a homosexual relationship during his teens and showed some perversions involving women. He used to meet prostitutes in the street, but only to ask them if they offered the kind of sex for which he was looking. He became excited thinking of sexual relationships with very old ladies or performing bizarre sexual acts, but he experienced guilt over these thoughts and desires.

During our first meeting with the patient, he was anxious, depressed and very insecure. He could not engage in any social interaction and was afraid of other people's judgment. He confessed that he engaged in deliberate self-harm almost daily (such as cutting or inserting needles under his skin) in order to reduce his deep anxiety, anger and dysphoria. A central feature of this patient was his suicidal intent as he always felt hopeless and depressed, unable to have friends, a girlfriend or sustained social interactions. He had never attempted suicide, but he had a detailed plan for killing himself. He intended to jump from a window if he experienced another serious depressive episode. He had guilt delusions based on the large amount of money spent for his eye treatments. He also had hypochondriac delusions apparently based on mild ailments which were later identified as side-effects of the medications that he was taking.

Another feature of his personality was somatic anxiety. His disorder distressed him in two totally different areas. On one hand, he felt excited by his desires and thoughts; on the other one hand he felt guilt over them and condemned them. This guilt led to anxiety and anger, resulting in deliberate self-harm and suicidality.

One of the authors (MP) treated him with regular sessions of psychotherapy. At the beginning the patient was reluctant to talk. He focused on his everyday difficulties, especially his belief of being stared by other people. After a few months of psychotherapy, the patient revealed important facts of his childhood life, especially related to his parents' behavior. His mother was described as cold and lacking feelings. The patient had experienced very strong hatred for his parents for which he felt guilty. This severe guilt led him to the eye injury. Contrary to expectations, the eye self-injury in our patient was not related to any religious belief. Psychotherapy also addressed his negative transference feelings which were always covered with politeness and compliance with the therapy.

At the time that he applied for a psychiatric consultation, he felt hopeless and helpless but highly motivated to start a new treatment. We prescribed quetiapine 800 mg a day, lamotrigine 200 mg a day and lithium carbonate 600 mg a day. We also gave him the chance to start psychodynamic psychotherapy with one or two sessions per week depending on factors such as his occasional request to meet therapist twice a week, suicidal crises or serious episodes of hopelessness.

After eighteen months, the patient had dramatically improved. Not only did he feel less depressed and more positive about the future, but he was able to talk about the eye injury without feeling guilty, recalling the stressful period during which he had injured his eye. He was also less suicidal, reporting thoughts of suicide only from time to time.

## Discussion

This patient had been seen by many psychiatrists, and most of them had showed a reluctance to engage in a sound patient-doctor relationship. He had, therefore, simply been prescribed different medications with no real improvement. Several psychiatrists had prescribed heavy doses of various psychotropic medication with no scientific rationale.

Suicide risk was a major issue in this patient especially during the boring and empty days when he was hopeless, unable to leave his home and finding no reason to continue living.

According to his description, the injury to his right eye was performed during one of these days in order to "change things" and "to feel the pain in the body and not in the mind." It was also during these moments that he wanted to commit suicide. The therapeutic alliance was a key feature with this patient. Treatment was tailored to his needs. Shneidman [17] conceptualized suicide as best understood, not so much as a movement toward death, but as a movement away from something which is always the same: intolerable emotion, unendurable pain or unacceptable anguish. If the level of suffering is reduced, the individual will choose to live. Profound psychic pain is a major part of the clinical picture of suicidal individuals, so much so that self-harming thoughts and behaviors, including self-mutilation, as well as suicidal ideation, gestures and attempts, may become a way of attempting to cope with this pain. The best way to prevent suicide is to learn what is causing the distress, the tension and the anguish.

Another key factor in this patient was the exclusion of antidepressants. In fact, it became clear that the agitation, insomnia, dysphoria and anger, as well as his suicidality, during his periods of depression were made worse by the antidepressants (both tricyclics and SSRIs) that he had been prescribed. Recent reports suggested that caution is imperative in prescribing antidepressants to people who are at risk of suicide or to those people who are vulnerable to develop suicidality as a result of antidepressant medications [18,19]. Nevertheless, generalizing about this risk is incorrect given the results of a recent meta-analysis [20] showing that antidepressants significantly reduce suicidal behavior in the vast majority of patients and increase such risk only in a very small vulnerable subpopulation. Also, when treating depressed patients clinicians should bear in mind the possibility of a misdiagnosed bipolar disorder. Benazzi [21] pointed out that depressed patients are often bipolar II patients, and he stressed the need to better distinguish between major depressive and bipolar disorders. Antidepressants may have a negative effect on the course of bipolar disorders, especially in the case of bipolar depression which is generally worsened by such treatment.

Patients who deliberately injure their eyes cause great distress to clinicians and often are avoided or treated pharmacologically in order to minimize contact with them. This feature is found also in the treatment of suicidal people. Both disorders require clinical skills and an opportunity for the patient to experience a solid patient-doctor relationship.
